# Editorial: Autoimmune and Inflammatory Rheumatic Diseases: Identifying Biomarkers of Response to Therapy With Biologics

**DOI:** 10.3389/fphar.2021.815656

**Published:** 2021-12-10

**Authors:** Anna Lisa Giuliani, Alessandra Bortoluzzi, Maria Efthymiou, Francesca Oliviero

**Affiliations:** ^1^ Department of Medical Sciences, University of Ferrara, Ferrara, Italy; ^2^ City of London College, London, United Kingdom; ^3^ Department of Medicine - DIMED, University of Padua, Padua, Italy

**Keywords:** autoimmune diseases, inflammatory rheumatic diseases, biomarkers, therapies, biologic drugs

The scope of this research topic was to provide an updated overview of the advances in the identification of new biomarkers of response to biologic drugs in autoimmune and inflammatory rheumatic diseases.

The use of biologic agents in autoimmune and inflammatory rheumatic diseases, has greatly increased in recent years, providing new treatment options to conventional anti-inflammatory and immunosuppressive strategies. Biologic drugs very often show selectivity of action towards specific targets and are currently used to induce disease remission or treat specific organ involvements in various inflammatory and autoimmune diseases. Given the variability in biological and clinical response between patients and the lack of knowledge in the mechanisms influencing individual responses, it has become urgent to identify biomarkers of response to therapy, especially to individuate patients who may potentially benefit from these therapies. This also in view of the possible role of biomarkers as indicators of personalized treatment. This research topic contains description of various diseases treated with biological drugs in 11 articles: 4 reviews, 1 systematic review, 5 original research papers, and 1 clinical trial.

Systemic lupus erythematosus (SLE) is one of the most severe among autoimmune rheumatic diseases characterized by complex and not well known pathogenesis and involvement of a high number of body organs. A study included in this research topic from Piantoni et al. (Front. Pharmacol. 2021 May 21; 12:666971) has reported the characterization of circulating peripheral B and T lymphocytes and the evaluation of soluble B-cell related factors belonging to the TNF/TNFR superfamily in a cohort of clinically active SLE patients treated with belimumab. It was found that the baseline BAFF serum levels are the strongest predictor of response to belimumab after 12 months of therapy.

Rheumatoid arthritis (RA) is another common autoimmune rheumatic disease characterized by involvement at joints and, in more severe cases, of different organs with increased mortality. RA has been dealt with in four papers of our research topic. A phase Ib/IIa clinical trial from Tang et al., has investigated the pharmacokinetics and pharmacodynamics of WBP216, a long half-life fully human monoclonal antibody against interleukin (IL)-6, in a limited number of Chinese RA patients to optimize the dosage regimen for future clinical trials (Front Pharmacol. 2021 Feb 18; 12:617265). Another study from Li et al. aimed to illustrate the regulation of Treg cells by arsenic trioxide (As_2_O_3_) in the pathogenesis of early RA. The researchers identified significantly modulated pathways and/or functional categories of genes regulating metabolism in Treg cells, a finding that might be important in the treatment-naïve RA (Front Pharmacol. 2021 May 24; 12:656124). Alternative approach with respect to conventional anti-inflammatory and immunomodulatory drugs is presented in the review from Cai et al. which summarized the roles of the main enzymes and their derivatives involved in the pathogenesis of RA and explored the explicit and potential targeted actions of herbal medicinal products with anti-RA activity (Front Pharmacol. 2021 Mar 16; 12:626342). A new perspective in RA treatment can be inferred by the data reported in the paper from Xiao et al. In this study, an animal model of RA, the collagen-induced arthritis (CIA) model, has been used to evaluate alterations in the gut microbial communities in the ileum and cecum of CIA and control rats including microbial richness, diversity and taxa as well as the expression of IL-1β and IL-17A in the ileum and cecum of CIA rats. The ileal microbiota of CIA rats presented main alterations together with significant increase in IL-1β and IL-17A mRNA expression, consistently with the immune-mediated inflammatory features of CIA (Fron Pharmacol. 2020 Nov 24; 11:587,534).

A review from Choida et al. (Front Pharmacol 2021 Feb 2; 11:635,823.) presented the results of a comprehensive search in the literature to identify clinical, serological, genetic, cellular, and imaging biomarkers that can assist clinicians in efforts to personalize disease-modifying anti-rheumatic drugs (DMARDs) prescription and adjust treatment strategies for Juvenile idiopathic arthritis (JIA) patients, with particular attention to studies investigating etanercept treatment in the most severe JIA phenotype. The conclusion of this study is that, although there are no ideal biomarkers in JIA, serological biomarkers with potential clinical utility have been identified and strategies of combining biomarkers of response to biologics in JIA is suggested.

A narrative review by Silvagni et al. (Front Pharmacol. 2021 Jun 15; 12:672515) provided an up-to-date overview of targeted therapies and treatment response biomarkers in psoriatic arthritis. This review focused on TNF-α-, IL-23/IL-17-, and JAK/STAT-dependent signal transduction axes, in the optics to define the relationship among different activated proinflammatory processes suitable for targeting by different currently available drugs.

Dermatomyositis (DM) is a rare autoimmune disease defined as an idiopathic inflammatory myopathy characterised by cutaneous manifestations. The study from Li et al. (Front Pharmacol. 2021 Sep 17; 12:727901), identified the profiles of noncoding RNAs, both lncRNAs and miRNAs, carried in exosomes (EXOs) from peripheral neutrophils of DM patients. This revealed a high number of upregulated and downregulated noncoding RNAs in DM neutrophil EXOs. The study also explored their potential functional roles and utility as new biomarkers and therapeutic targets.

A study from Lu et al. (Front Pharmacol. 2021 Mar 16; 12:635654) has evaluated the efficacy of tocilizumab (TCZ) in adult patients with refractory immune-mediated necrotizing myopathies and investigated possible predictive biomarkers of the response to treatment with TCZ. Baseline serum IL-6 and muscle IL-6 mRNA levels and the percentage of CD56-positive muscle fibres have been found useful to predict the response to TCZ treatment in these patients.

Non-infectious uveitis (NIU) is believed to be an immune-mediated ocular inflammation frequently associated to systemic autoimmune diseases such as JIA. The systematic review from Li et al. (Front Pharmacol. 2021 Apr 26; 12:673984) summarised the current evidence from randomised controlled trials regarding the efficacy and safety of adalimumab (ADA) treatment in NIU. Metanalysis performed on seven randomized controlled trials (RCTs) confirmed that ADA considerably lowered the risk of treatment failure or visual loss as compared to placebo, leading to the conclusion that ADA is both effective and safe in treating NIU.

Biologic therapies are employed in many inflammatory and immune mediated diseases, such as psoriasis, a chronic inflammatory disease characterised by erythematous scaly plaques frequently accompanied by systemic damages. The review from Shi et al. explored the most life-threatening associations between psoriasis and cardiometabolic comorbidities (cardiovascular diseases, obesity, diabetes mellitus, and metabolic syndrome), emphasizing benefits and precautions of biologic therapy in the management of psoriatic patients with cardiometabolic comorbidities and outlining the positive effects of different biologics on cardiovascular biomarkers (Front Pharmacol. 2021 Nov 4;12:774808).

In conclusion, this research topic explored and summarised many different aspects of therapies with biological treatments in inflammatory and immune mediated diseases. The effects of monoclonal antibodies (belimumab, ADA, TCZ, etanercept, and WBP216) as well as chemical compounds (arsenic trioxide) and medicinal herbs have been evaluated in addition to the investigation of different aspects influencing autoimmune and inflammatory diseases ranging from non-coding RNA in EXOs to gut microbial communities’ activity.

